# Overexpression of Parkinson’s Disease-Associated Mutation LRRK2 G2019S in Mouse Forebrain Induces Behavioral Deficits and α-Synuclein Pathology

**DOI:** 10.1523/ENEURO.0004-17.2017

**Published:** 2017-03-17

**Authors:** Yulan Xiong, Stewart Neifert, Senthilkumar S. Karuppagounder, Jeannette N. Stankowski, Byoung Dae Lee, Jonathan C. Grima, Guanxing Chen, Han Seok Ko, Yunjong Lee, Debbie Swing, Lino Tessarollo, Ted M. Dawson, Valina L. Dawson

**Affiliations:** 1 Neuroregeneration and Stem Cell Programs, Institute for Cell Engineering, Johns Hopkins University School of Medicine, Baltimore, Maryland 21205; 2 Department of Neurology, Johns Hopkins University School of Medicine, Baltimore, Maryland 21205; 3 Soloman H. Snyder Department of Neuroscience, Johns Hopkins University School of Medicine, Baltimore, Maryland 21205; 4 Department of Physiology, Johns Hopkins University School of Medicine, Baltimore, Maryland 21205; 5 Department of Pharmacology and Molecular Sciences, Johns Hopkins University School of Medicine, Baltimore, Maryland 21205; 6 Adrienne Helis Malvin Medical Research Foundation, New Orleans, Louisiana 70130; 7 Diana Helis Henry Medical Research Foundation, New Orleans, Louisiana 70130; 8 Department of Anatomy and Physiology, Kansas State University College of Veterinary Medicine, Manhattan, Kansas 66506; 9 Neural Development Section, Mouse Cancer Genetics Program, Center for Cancer Research, National Cancer Institute, Frederick, Maryland 21702

**Keywords:** Parkinson’s disease, LRRK2, transgenic mice, α-synuclein

## Abstract

Mutations in the leucine-rich repeat kinase 2 (LRRK2) gene have been identified as an unambiguous cause of late-onset, autosomal-dominant familial Parkinson’s disease (PD) and LRRK2 mutations are the strongest genetic risk factor for sporadic PD known to date. A number of transgenic mice expressing wild-type or mutant LRRK2 have been described with varying degrees of LRRK2-related abnormalities and modest pathologies. None of these studies directly addressed the role of the kinase domain in the changes observed and none of the mice present with robust features of the human disease. In an attempt to address these issues, we created a conditional LRRK2 G2019S (LRRK2 GS) mutant and a functionally negative control, LRRK2 G2019S/D1994A (LRRK2 GS/DA). Expression of LRRK2 GS or LRRK2 GS/DA was conditionally controlled using the tet-off system in which the presence of tetracycline-transactivator protein (tTA) with a CAMKII*α* promoter (CAMKII*α*-tTA) induced expression of TetP-LRRK2 GS or TetP-LRRK2 GS/DA in the mouse forebrain. Overexpression of LRRK2 GS in mouse forebrain induced behavioral deficits and α-synuclein pathology in a kinase-dependent manner. Similar to other genetically engineered LRRK2 GS mice, there was no significant loss of dopaminergic neurons. These mice provide an important new tool to study neurobiological changes associated with the increased kinase activity from the LRRK2 G2019S mutation, which may ultimately lead to a better understanding of not only the physiologic actions of LRRK2, but also potential pathologic actions that underlie LRRK2 GS-associated PD.

## Visual Abstract

**Figure:**
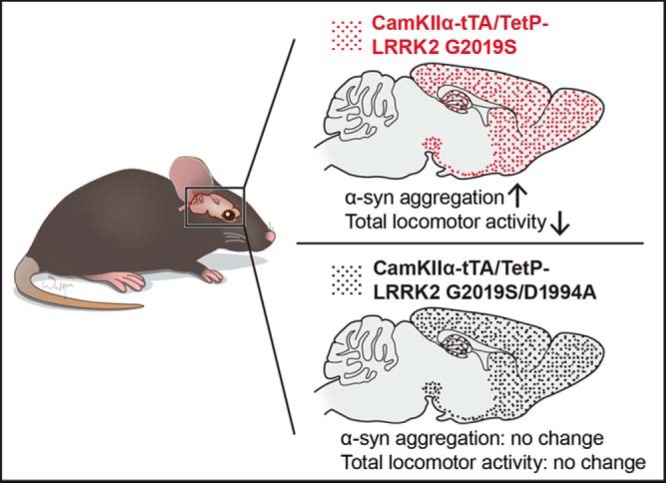

## Significance Statement

Mutations in leucine-rich repeat kinase 2 (LRRK2) gene are the most common genetic cause for both familial and sporadic Parkinson’s disease to date with the G2019S LRRK2 (LRRK2 GS) being the most prevalent mutation. The clinical presentation of patients carrying LRRK2 GS is indistinguishable from sporadic disease in many cases. Many lines of evidence indicate that LRRK2 GS has increased kinase activity and that *in vitro* LRRK2 inhibitors or kinase-dead G2019S/D1994A double mutants (LRRK2 GS/DA) reduce LRRK2 GS-mediated toxicity, indicating that LRRK2 associated toxicity is kinase dependent. However, this concept remains controversial. To address this question *in vivo*, we developed a new tet-inducible conditional transgenic LRRK2 GS and LRRK2 GS/DA mouse model, which exhibits behavioral deficits and α-synuclein pathology in a kinase-dependent manner.

## Introduction

Parkinson’s disease (PD) is recognized as the most common movement disorder. The cardinal symptoms are caused by the progressive degeneration of dopaminergic (DA) neurons in the substantia nigra pars compacta (SNpc; Lees et al., 2009). Mutations in the leucine-rich repeat kinase 2 (*LRRK2*) gene have been linked to both familial and sporadic forms of PD (Paisán-Ruíz et al., 2004; Zimprich et al., 2004). The LRRK2 protein contains two enzymatic domains (GTPase and kinase domains) and multiple protein–protein interacting domains including an LRR, a WD40 repeat, and a LRRK2-specific repeat domain (Mata et al., 2006; Cookson, 2010). The G2019S (GS) mutation within the kinase domain is the most common mutation of LRRK2, which alters LRRK2 GTPase and kinase activities (Lees et al., 2009; Martin et al., 2014b). LRRK2 GS mutations lead to alterations in vesicle trafficking, neurite outgrowth, autophagy, cytoskeletal dynamics *in vitro* (Martin et al., 2014b; Cookson, 2015), as well as defects in protein translation both *in vitro* and *in vivo* (Imai et al., 2008; Gehrke et al., 2010; Dorval and Hébert, 2012; Martin et al., 2014a,b,c). To study the function of LRRK2 and potential pathologic actions of LRRK2 mutations, many lines of transgenic (Tg) or knock-in mice have been generated. These animal models have provided interesting and valuable biochemical and physiologic insights, but none of these models have recapitulated key features of PD (Li et al., 2009, 2010; Lin et al., 2009; Tong et al., 2009; Melrose et al., 2010; Herzig et al., 2011; Ramonet et al., 2011; Chen et al., 2012; Tsika et al., 2014; Beccano-Kelly et al., 2015; Garcia-Miralles et al., 2015; Liu et al., 2015; Volta et al., 2015; Yue et al., 2015; Weng et al., 2016). Interestingly, no animal models have been created to directly test the role of increased kinase activity with the common LRRK2 GS mutation. To address this, we developed a new Tet-inducible conditional LRRK2 mutant G2019S (TetP-LRRK2 GS) transgenic mouse line and a corresponding functionally negative control G2019S/D1994A (TetP-LRRK2 GS/DA) mouse line under the control of a tetracycline-responsive regulator. Crossbreeding the TetP-LRRK2 GS or TetP-LRRK2 GS/DA mice to CamKII*α*-tTA mice (Mayford et al., 1996) led to high expression levels of LRRK2 GS or the control LRRK2 GS/DA in adult mouse forebrain. High expression of LRRK2 GS induces behavioral deficits and α-synuclein pathology but does not cause significant dopamine neurodegeneration. Nevertheless, these novel transgenic mice offer an excellent platform to explore and elucidate the kinase-dependent actions of LRRK2 in the brain.

## Materials and Methods

### Animals

Mice were housed and treated in accordance with the National Institutes of Health (NIH) *Guide for the Care and Use of Laboratory Animals* and Institutional Animal Care and Use Committees. Animals were housed in a 12 h dark/light cycle with free access to water and food. Both male and female animals were assigned to groups by computer-generated randomization for all experiments. Mice were acclimatized for 3 d in the procedure room before any experiments were started. Sample size was justified by power analysis.

### Generation of conditional *LRRK2* transgenic mouse

**Figure 1.**
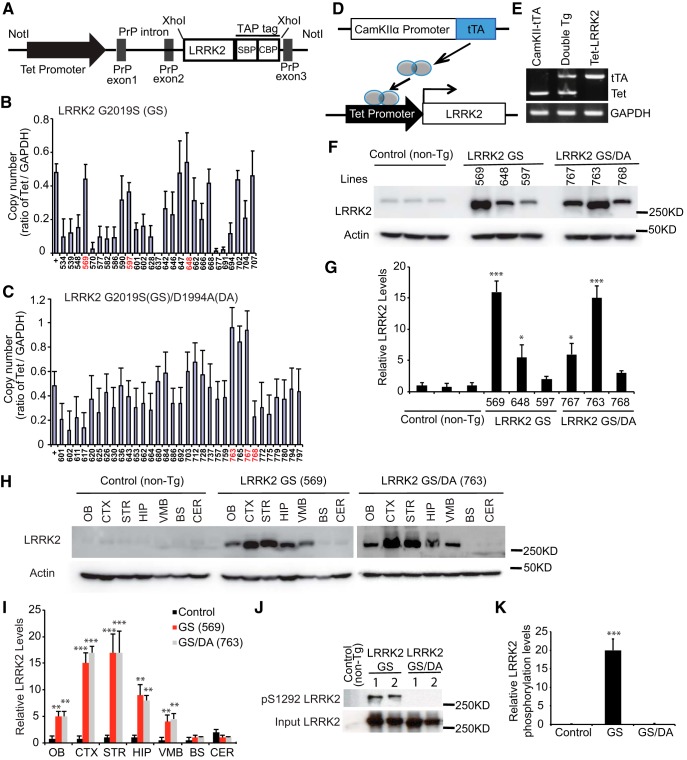
Generation of TET-inducible conditional LRRK2 transgenic mice. ***A***, Schematic diagram of the TetP-LRRK2-TAP construct. ***B***, Relative transgene copy number determined by semiquantitative PCR performed on genomic DNA expressed in arbitrary units as the ratio of the TetP-LRRK2-G2019S (GS) transgene to GAPDH for each founder mouse. ***C***, Relative transgene copy number determined by semiquantitative PCR performed on genomic DNA expressed in arbitrary units as the ratio of the TetP-LRRK2-GS2019S/D1994A (GS/DA) transgene to GAPDH for each founder mouse. ***D***, Schematic diagram of the generation of LRRK2-inducible transgenic mice using the “tet-off” system. ***E***, Representative genotyping PCR for TetP-LRRK2 and CamKIIα-tTA using genomic DNA. GAPDH PCR was used as an internal control. ***F***, Western blot analysis of LRRK2 expression from LRRK2 transgenic mouse brain. Each number represents a single LRRK2 transgenic founder line: 569, 648 and 597 of LRRK2-GS, 767, 763, and 768 LRRK2-GS/DA transgenic mice. ***G***, Quantification of LRRK2 expression in mouse brains normalized to β-actin, *n* = 3. Differences between transgenic and control groups were assessed by unpaired, two-tailed Student’s *t* test. Quantified data are expressed as the mean ± SEM. *p < 0.05, ***p < 0.001. ***H***, Representative Western blots of LRRK2 distribution in brain subregions from control and LRRK2-GS (line 569) and LRRK2-GS/DA (line 763) transgenic mice (OB; CTX; STR; HIP; VMB; BS; CER). ***I***, Quantification of LRRK2 distribution in mouse brains normalized to β-actin, *n* = 3. ***J***, Western blot analysis of total protein from mouse brains of control, LRRK2 GS, and LRRK2 GS/DA by anti-LRRK2 and LRRK2 phosphor S1292 antibodies. *K*, Quantification of phosphor Ser1292 LRRK2 levels in mouse brains normalized to total LRRK2 protein level, *n* = 3. Quantified data are expressed as the mean ± SEM: *p < 0.05, **p < 0.01, ***p < 0.001, Differences between transgenic and control groups were assessed by two-way ANOVA. There was a nonsignificant difference between LRRK2 GS and LRRK2 GS/DA groups.

A tandem affinity purification (TAP) tag composed of the streptavidin-binding peptide and calmodulin-binding peptide was cloned into C-terminal human LRRK2 GS or a LRRK2 GS/DA under the control of a tetracycline responsive regulator (Fig. 1*A*). The transgenic constructs were linearized by the NotI enzyme and subsequently microinjected into the embryos of B6C3F2 mice. One- or two-cell embryos were transferred into B6D2F1 pseudopregnant female mice. Genomic DNA was prepared from tail snip (Proteinase K, Roche Diagnostics; direct PCR tail Lysis, Viagen), and pups were genotyped by PCR (DreamTaq Green Master Mix, Thermo Scientific) using *TetP-LRRK2* primers (forward, CGG GTC GAG TAG GCG TGT AC; reverse, TCT AGA TGA TCC CCG GGT ACC GAG; PCR product = 173 bp). Positive founders were selected and further subjected to semiquantitative PCR and normalized to GAPDH PCR (forward, AAA CCC ATC ACC ATC TTC CAG; reverse, AGG GGC CAT CCA CAG TCT TCT; PCR product = 300 bp) to screen for high-copy number founders. The three highest copy number founders were selected and bred with C57BL/6 mice to generate F1 progeny and to establish the transgenic lines. The following primer sets were used for genotyping of *CamKIIα-tTA* (forward, TGA AAG TGG GTC CGC GTA C; reverse, TAC TCG TCA ATT CCA AGG GC; PCR product = 391 bp). LRRK2 induction in conditional transgenic mice was suppressed by feeding the mice with doxycycline-containing food (doxycycline Diet-Sterile, 200 mg/kg doxycycline, Bio-Serv).

### Stereological assessment of the number of tyrosine hydroxylase- and Nissl-positive cells

Mice were perfused with ice-cold PBS, followed by 4% paraformaldehyde/PBS, pH 7.4. Brains were removed and postfixed overnight in the same fixative. After cryoprotection in 30% sucrose/PBS, brains were frozen on dry ice, and serial coronal sections (40 µm sections) were cut with a microtome. Every four sections were collected for subsequent procedures. Free-floating sections were blocked with 4% goat serum (Sigma-Aldrich)/PBS plus 0.3% Triton X-100 and incubated with antibodies to tyrosine hydroxylase (TH; rabbit polyclonal; Novus Biologicals; RRID:AB_1218296) followed by incubation with biotin-conjugated antibody to rabbit, ABC reagents (Vector Laboratories) and Sigmafast diaminobenzidine tablets (Sigma-Aldrich). Sections were counterstained with Nissl (0.09% thionin) after tyrosine hydroxylase staining, as previously described (Lee et al., 2013; Karuppagounder et al., 2016). Sections were dehydrated in 100% ethanol and cleared in xylene (Fisher Scientific) followed by mounting with DPX (Sigma-Aldrich) before imaging under a microscope. TH-positive and Nissl-positive DA neurons from the SNpc region were counted through an optical fractionator, the unbiased method for cell counting. This unbiased stereological counting was conducted by a computer-assisted image analysis system consisting of an Axiophot photomicroscope (Carl Zeiss Vision) equipped with a computer-controlled motorized stage (Ludl Electronics), a Hitachi HV C20 video camera and Stereo Investigator software (MicroBrightField). Fiber density in the striatum was quantified by optical density (OD). ImageJ software (NIH) was used to analyze the OD as previously described (Karuppagounder et al., 2016).

### Western blotting

Brain extracts from indicated genotype were prepared by homogenization in lysis buffer [1× phosphate-buffered saline, 1% Triton X-100, 1× Complete protease inhibitor (4693116001, Sigma-Aldrich), 1× PhosSTOP phosphatase inhibitor (4906845001; Sigma-Aldrich)]. Protein concentration was determined by the BCA method (Pierce Biotechnology). Approximately 100 µg of protein was resolved by SDS-PAGE, transferred to polyvinylidene fluoride membrane and probed with mouse anti-LRRK2 antibody (N136/8, NeuroMab; RRID:AB_2234791) or rabbit anti-LRRK2 antibody (1304; D18E12, Cell Signaling Technology) recognizing both mouse and human LRRK2. Membranes were also probed with rabbit anti-LRRK2 phospho Ser1292 antibody (ab203181; MJFR-19-7-8, Abcam), mouse anti-α-synuclein antibody (610787; BD Transduction Laboratories; RRID:AB_398108), rabbit anti-α-synuclein antibody (2642; Cell Signaling Technology; RRID:AB_2192679), anti-actin-peroxidase rabbit polyclonal antibody for loading control (A3854; Sigma-Aldrich; RRID:AB_262011). Densitometric analysis was conducted to quantify the fold overexpression of LRRK2 relative to endogenous mouse LRRK2. Total LRRK2 protein levels were normalized to actin and expressed as the percentage of non-Tg controls. Mean values from three mice per genotype/control were analyzed for statistical significance by two-tailed unpaired Student’s *t* test compared with non-Tg controls.

### Open field test and d-amphetamine administration

Spontaneous locomotor and exploratory activities were assessed in open field square-shaped (16 × 16) chambers equipped with an automated photobeam tracking system (San Diego Instruments). Briefly, a mouse was placed in the center of the open field arena and allowed to explore the area for 25 min, following by d-amphetamine injection (7 mg/kg, s.c.; A-5880, lot #34H0145, Sigma-Aldrich) and another 25 min exploration. The activities of a mouse were recorded every minute by Photobeam Activity System software installed on a computer connected to the open field equipment. Before and after each testing, the clear acrylic enclosure of the surface of the arena was cleaned with 70% ethanol. The total number of beam breaks during the total 50 min period was used to determine gross locomotor activity of a mouse.

### Rotarod test

Motor coordination of mice was measured as the retention time on an accelerating rotarod of the rotamex V instrument equipped with photobeams and a sensor to automatically detect mice that fell from the rotarod (Columbus Instruments). Before the actual test, the mice were trained on the rotarod at 4.0–40 rpm for 5 min and allowed to rest for at least 30 min. The training occurred over 3 consecutive days and consists of three test trials. On the day of the test, four mice were placed on separate rods and the durations on the accelerating rods were recorded automatically by the software installed on a computer connected to the instrument. The setting of the rotamex was as follows: start speed, 4.0 rpm; maximum speed, 40 rpm; acceleration interval, 30 s; acceleration step, 4 rpm. The setting remained constant throughout all trials. The tests were blinded and evaluated in three sessions, and the average retention time and end speed were recorded for each mouse. The retention time was used to determine the motor coordination of the mouse.

### Pole test

The pole consists of a 2.5 foot metal rod with a 9 mm diameter that is wrapped with bandage gauze. Briefly, the mice were placed 3 inches from the top of the pole facing head-up. Total time taken to turn and reach the base of the pole was recorded. Before the actual test, the mice were trained for 3 consecutive days, and each training session consisted of three test trials. On the day of the test, mice were evaluated in three sessions with 1 h intervals in between, and total times were recorded. Results were expressed in total time in seconds (Karuppagounder et al., 2016)

### Statistical analysis

Two-way ANOVA and two-tailed unpaired Student’s *t* test was used for data analysis. Data represent **the** mean ± SEM, and *p* ≤ 0.05 was considered statistically significant: *p < 0.05, **p < 0.01, ***p < 0.001. Power analysis was performed by using G*Power 3.1 software to determine approximate sample sizes for behavior tests or stereological analysis.

## Results

### Generation of TET-inducible conditional LRRK2 transgenic mice

We generated tetracycline responsive transgenic mice (TetP-LRRK2 GS or TetP-LRRK2 GS/DA) using C-terminal TAP tagged human LRRK2 GS or LRRK2 GS/DA under the control of a tetracycline responsive regulator (Fig. 1*A*). Twenty-seven TetP-LRRK2 GS founders and 33 TetP-LRRK2 GS/DA founder mice were identified by PCR screening for the tetracycline promoter (Fig. 1*B*,*C*). Three male mice with the highest copy number were selected as founders and crossbred to CamKII α-tTA transgenic mice (Mayford et al., 1996; Fig. 1*D*). Dams were maintained on doxycycline food until pups were weaned to prevent expression of transgenes and possible compensation during development. Mice expressing both CamKII α-tTA and TetP-LRRK2 GS or TetP-LRRK2 GS/DA were identified by PCR (Fig. 1*E*). At ∼2 months of age, doxycycline food was withdrawn and the overexpression of LRRK2 GS or LRRK2 GS/DA was monitored. LRRK2 GS is overexpressed 16-, 5-, and 2-fold in lines 569, 648, and 597, respectively, and LRRK2 GS/DA is overexpressed 6-, 15-, 3-fold in lines 767, 763, and 768, respectively (Fig. 1*F*,*G*). Since line 569 and line 763 overexpress LRRK2 at similar levels, they were selected for further study. A regional assessment of the overexpression of LRRK2 GS (line 569) and LRRK2 GS/DA (line 763) were monitored by Western blot analysis (Fig. 1*H*,*I*). LRRK2 GS or GS/DA is overexpressed ∼5-, 15-, 17-, 9-, and 4-fold in the olfactory bulb (OB), cortex (CTX), striatum (STR), hippocampus (HIP), and ventral midbrain (VMB), respectively, while there is no significant change in LRRK2 expression in the brainstem (BS), and cerebellum (CER; Fig. 1*H*,*I*). Collectively, LRRK2 was induced at very high levels in the mouse forebrain. To monitor LRRK2 kinase activity and confirm that LRRK2 GS/DA is kinase dead *in vivo*, the phosphorylation status of LRRK2 was examined in LRRK2 GS and LRRK2 GS/DA mice by LRRK2 phosphor S1292 antibody (Fig. 1*J*,*K*). Western blots of total protein from mouse brains of control, LRRK2 GS, and LRRK2 GS/DA revealed that LRRK2 GS has a high phosphorylation level and LRRK2 GS/DA does not have a detectable kinase activity (Fig. 1*J*,*K*).

### Behavioral deficits of conditional LRRK2-G2019S transgenic mice

**Figure 2.**
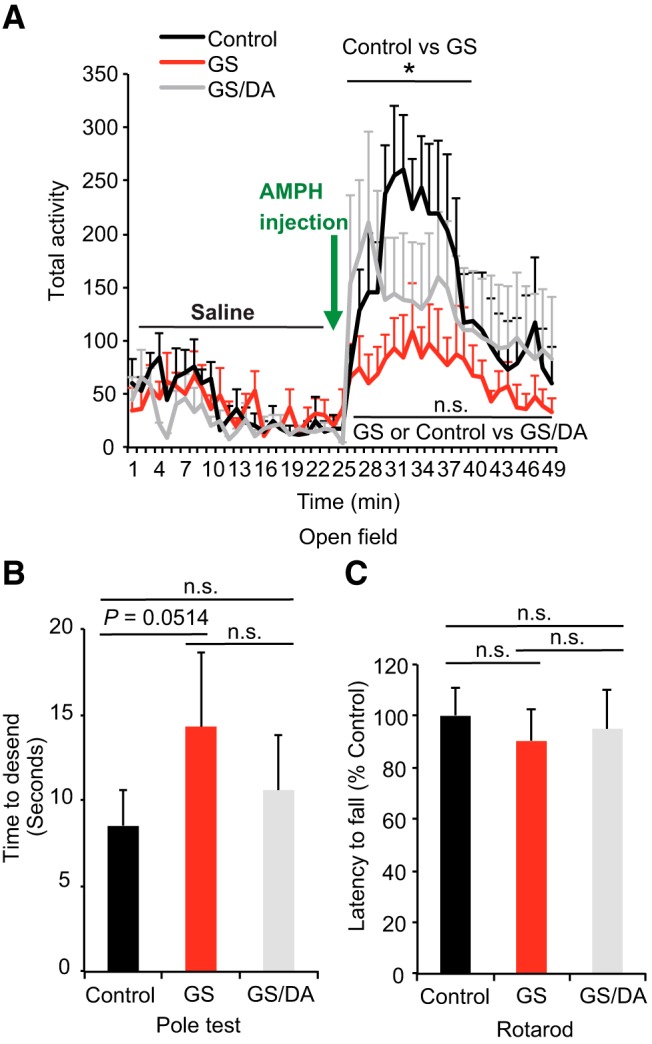
Behavioral deficits of conditional LRRK2-G2019S transgenic mice. ***A***, Open field analysis under normal conditions and following amphetamine challenge. Mice were placed in the center of the open field arena and allowed to explore the area for 25 min, following by d-amphetamine injection (7 mg/kg, s.c.) and another 25 min exploration. The total activities of mice were recorded every minute (control, *n* = 7; GS, *n* = 9; GS/DA, *n* = 8). ***B***, Pole test to monitor the behavioral abnormalities of 22-month-old LRRK2 GS and GSDA transgenic and age-matched littermate controls (control, *n* = 8; GS, *n* = 8; GS/DA, *n* = 7). ***C***, Assessment of latency to fall in an accelerated rotarod test (control, *n* = 8; GS, *n* = 9; GS/DA, *n* = 8). Data are the mean ± SEM. Statistical significance was determined by two-way ANOVA. *p < 0.05. n.s., Nonsignificant.

The potential effects of LRRK2 GS expression on motor behavior were assessed. Open field, pole, and rotarod testing were performed with LRRK2 GS and LRRK2 GS/DA transgenic mice. LRRK2 GS and LRRK2 GS/DA transgenic mice performed similarly as nontransgenic control mice under normal conditions in the open field test at 10, 15, and 22 months of age (Fig. 2*A*; data not shown). There was no significant difference between LRRK2 GS and LRRK2 GS/DA at 10 and 15 months of age (data not shown). Interestingly, LRRK2 GS mice have a blunted response to d-amphetamine administration (7 mg/kg, s.c.) at 22 months, whereas nontransgenic and LRRK2 GS/DA mice exhibit increased activity (Fig. 2*A*). In the dopamine-sensitive pole test assessed at 22 months of age, LRRK2 GS transgenic mice had an insignificant deficit in ability to descend the pole (*p* = 0.0514). The LRRK2 GS/DA performed similar to nontransgenic control mice (Fig. 2*B*). In the rotarod test, both the LRRK2 GS and LRRK2 GS/DA transgenic mice performed normally at 10, 15, and 22 months of age (Fig. 2*C*; data not shown). Together, these results indicate that the LRRK2 GS mice have no robust DA-sensitive behavioral deficits.

### Conditional LRRK2-G2019S transgenic mice exhibit normal nigrostriatal dopaminergic pathway

**Figure 3.**
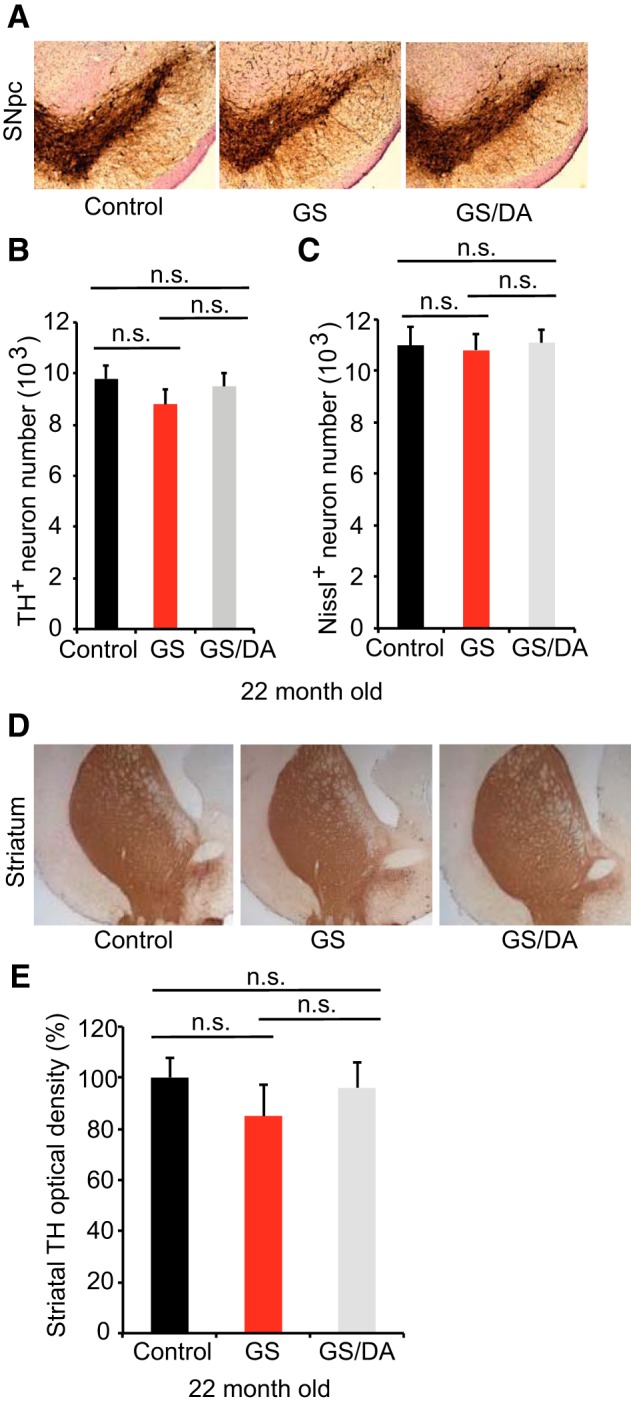
Characterization of the nigrostriatal pathway of LRRK2 conditional transgenic mice. ***A***, Representative TH immunohistochemistry of the midbrain coronal sections of 22-month-old LRRK2 GS and GSDA transgenic and age-matched littermate controls. ***B***, ***C***, Stereological assessment of TH-positive (***B***) and Nissl-positive (***C***) neurons in the SNpc (control, *n* = 9; GS, *n* = 9; GS/DA, *n* = 9). Data are the mean number of cells per region ± SEM, *n* = 9 mice per group. Statistical significance was determined by two-tailed unpaired Student’s *t* test. ***D***, Representative images of TH immunostaining of nerve terminals in the striatum of LRRK2 conditional transgenic mice at 22 months of age. ***E***, Quantitation of TH immunostaining in the striatum using ImageJ software (NIH; control, *n* = 7; GS, *n* = 7; GS/DA, *n* = 7). Differences between groups were assessed by two-way ANOVA. Bars represent the mean ± SEM (*n* ≥ 5 animals/genotype). n.s., Nonsignificant.

To determine whether temporal overexpression of LRRK2 GS in the mouse forebrain induces the degeneration of midbrain DA neurons, we assessed DA neuronal number by unbiased stereological counting of TH- and Nissl-positive neurons at 22 months of age. No significant DA neuronal loss was observed (Fig. 3*A–C*). Dopaminergic neurons are of normal size and morphology in the substantia nigra of LRRK2 GS mice (Fig. 3*A*). The fiber density of TH-positive dopaminergic nerve terminals in the striatum are unaltered in LRRK2 GS mice at 22 months of age (Fig. 3*D*,*E*). Together, our data suggest that the high levels of expression of LRRK2 GS in mouse forebrain are not sufficient to induce the degeneration of dopaminergic neurons of the nigrostriatal dopaminergic pathway with advancing age. These findings are consistent with the behavioral data.

### α-Synuclein pathology in conditional LRRK2-G2019S transgenic mice

**Figure 4.**
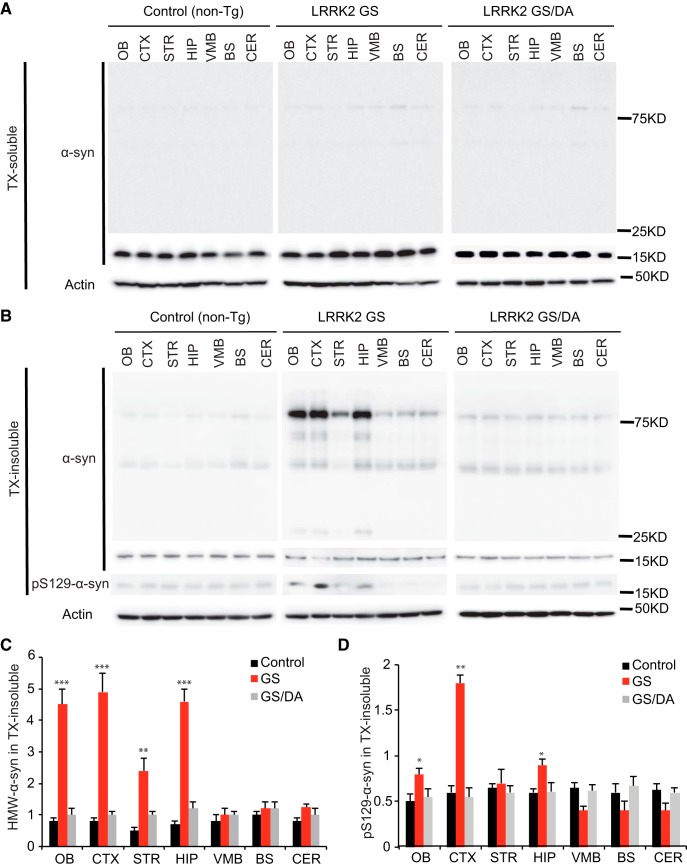
The levels of α-synuclein aggregation in the LRRK2 GS and LRRK2 GSDA mice. ***A***, Representative immunoblots of α-synuclein and β-actin in the Triton X-100 (TX)-soluble fraction of different brain regions from 22-month-old transgenic mice and age-matched littermate non-Tg controls. ***B***, Representative immunoblots of α-synuclein and β-actin in the TX-insoluble fraction of different brain regions from 22-month-old transgenic mice and age-matched littermate non-Tg controls. In the insoluble fractions, high-molecular-weight (75 kDa) species of α-synuclein are detected in OB, CTX, STR, and HIP but not in VMB, BS, and CER of LRRK2 GS mice. ***C***, Quantification of high-molecular-weight (HMW) α-synuclein protein levels in ***B*** normalized to β-actin (control, *n* = 3; GS, *n* = 3; GS/DA, *n* = 3). ***D***, Quantification of pS129 α-synuclein protein levels in ***B*** normalized to α-synuclease monomer (17 kDa; control, *n* = 3; GS, *n* = 3; GS/DA, *n* = 3). Differences between LRRK2 GS and the control or LRRK2 GS/DA group were assessed by two-way ANOVA. Bars represent the mean ± SEM. *p < 0.05, **p < 0.01, ***p < 0.001. There was a nonsignificant difference between LRRK2 GS/DA and control groups.

Since the majority of patients carrying the LRRK2 GS mutation have α-synuclein positive Lewy bodies and increased levels of phospho-serine 129 (pS129) α-synuclein, the status of endogenous α-synuclein was monitored in the nontransgenic control, LRRK2 GS, and LRRK2 GS/DA mice. Immunoblot analysis for α-synuclein in the Triton X-100-soluble fraction reveals no substantial difference in the immunoreactivity for α-synuclein (Fig. 4*A*) and no detectable immunoreactivity for pS129 α-synuclein (data not shown) at 22 months between LRRK2 GS mice and LRRK2 GS/DA mice. In the Triton X-100-insoluble fraction, the observed high-molecular-weight species (>75 kDa) of α-synuclein are indicative of aggregation and are similar between LRRK2 GS and LRRK2 GS/DA mice in the VMB, BS, and CER regions but are significantly increased in the OB, CTX, STR, and HIP of LRRK2 GS mice at 22 months of age compared with LRRK2 GS/DA and non-Tg control mice (Fig. 4*B*,*C*). Moreover, in the Triton X-100-insoluble fraction, low and similar levels of immunoreactivity for pS129 α-synuclein are observed in STR, VMB, BS, and CER brain regions of non-Tg, LRRK2 GS, and LRRK2 GS/DA mice. In contrast, pS129 α-synuclein levels are significantly increased in OB, CTX, and HIP brain regions of 22-month-old LRRK2 GS mice compared with LRRK2 GS/DA and non-Tg control mice of the same age (Fig. 4*B*,*D*). No significant difference in the immunoreactivity for α-synuclein and pS129 α-synuclein in both Triton X-100-soluble and insoluble fractions was observed at the ages of 10 and 15 months among LRRK2 GS, GS/DA, and non-Tg control mice (data not shown). This observation suggests that the LRRK2 GS mutation can promote α-synuclein pathology in a kinase- and age-dependent manner.

## Discussion

It is well established that the disease causing LRRK2 GS mutation exhibits increased kinase activity for both autophosphorylation and hyperphosphorylation of LRRK2 kinase substrates, while LRRK2 kinase inhibitors or kinase-dead G2019S/D1994A double mutants reduce LRRK2 GS-mediated toxicity indicating that LRRK2 toxicity is kinase dependent (Martin et al., 2014b; Cookson, 2015). This conclusion remains controversial, and alternative hypotheses have been suggested (Skibinski et al., 2014). To define the kinase-dependent and kinase-independent pathophysiologic actions of LRRK2, additional models are needed. Thus, we generated a conditional tet-off LRRK2 G2019S (LRRK2 GS) mutant and a functionally negative control, LRRK2 G2019S/D1994A (LRRK2 GS/DA), driven by the CAMKIIα promoter. Overexpression of LRRK2 GS in mouse forebrain induced behavioral deficits and α-synuclein pathology in a kinase-dependent manner, whereas these events were absent in the LRRK2 GS/DA mice. However, consistent with other genetically engineered LRRK2 GS mice, there was no significant loss of dopaminergic neurons.

Since the majority of LRRK2 PD patients exhibit α-synuclein aggregation, the role of LRRK2 in α-synuclein pathology in different LRRK2 mouse models has been explored. Lin et al. (2009) showed that overexpression of LRRK2 in mouse forebrain promotes the abnormal aggregation of exogenously overexpressed α-synuclein and knockout of LRRK2-rescued A53T α-synuclein overexpression-induced abnormalities. However, Tong et al. (2010) demonstrated that LRRK2 knock-out mice present with a robust aggregation of α-synuclein, while Daher et al. (2012) showed that knockout of LRRK2 has no influence on A53T α-synuclein-induced neurodegeneration. The different findings between these studies could be due to the different α-synuclein expression levels or technical concerns. Our LRRK2 mouse model provides the first evidence that the overexpression of LRRK2 in mouse forebrain induces endogenous α-synuclein aggregation and increased pS129 α-synuclein levels, which occur in a kinase-dependent manner. Whether LRRK2 could be used as a therapeutic target for α-synuclein-mediated neurodegeneration remains to be elucidated.

The reasons why past rodent models of the LRRK2 GS mutation as well as our new transgenic mouse model do not exhibit nigral neurodegeneration is not known, although many α-synuclein-based mouse models also lack nigral neurodegeneration (Lee, et al., 2012). Possibilities include compensatory mechanisms, levels of expression that are higher in brain regions other than the ventral midbrain or substantia nigra, poor expression in the substantia nigra, or lack of expression of the LRRK2 GS mutation in cell types other than neurons such as astrocytes or microglia.

All of these possibilities should be taken into consideration in developing new LRRK2 animal models meant to study neurodegeneration. The lack of neurodegeneration does not diminish the value of these models for biochemical investigations of the function of LRRK2. When coupled with the ability to purify the LRRK2 protein complex via the TAP tag, our new mouse model permits the investigation of increased kinase activity on LRRK2 biologic substrates and outcomes. Overall, these mice provide an important new tool to study neurobiologic changes that are due to the overactivation of the kinase activity by the LRRK2 GS mutation, which may lead to a better understanding of not only the physiologic functions of LRRK2, but also the potential pathologic mechanisms underlying LRRK2 GS-associated PD.

## Synthesis

Reviewing Editor: Julie Andersen, Buck Institute Decisions are customarily a result of the Reviewing Editor and the peer reviewers coming together and discussing their recommendations until a consensus is reached. When revisions are invited, a fact-based synthesis statement explaining their decision and outlining what is needed to prepare a revision will be listed below. The following reviewer(s) agreed to reveal their identity: Bobby Thomas, David Lynch.

Both reviewers agreed that the work describes a novel LRRK model which displays phenotypes of relevance to human disease and therefore is an important advance to the field. Some questions and technical improvements have been suggested below by the reviewers:

Reviewer #1:

Mutations in LRRK2 gene are the most common familial form of PD. Authors in the present study generated and characterized conditional tet-off LRRK2 transgenic mice under a tTA-CamKinase promoter system consisting of the kinase mutant (LRRK2-G2019S) and kinase dead mutant (LRRK-G2019S/D1994A). They show that overexpression of LRRK2-G2019S in mouse forebrain induced amphetamine-induced behavioral deficits and alpha-synuclein oligomers formation, without loss of nigrostriatal DA neurons in a kinase dependent manner as these events were absent in the kinase mutant LRRK2-G2019S/D1994A mice. They conclude that the observed behavioral deficits and alpha-synuclein abnormalities are directly associated with overactivation of the LRRK2 kinase activity.

Weakness-

1. It is unclear if amphetamine-induced behavioral deficits and alpha-synuclein pathology can also be observed in younger mice in a kinase dependent fashion. No rationale is provided why only 22-month-old mice were used for all the experiments in the manuscript.

2. Fig 1H: Which LRRK2 transgenic mice are used? LRRK2GS or LRRK2GSDA? Also mention the founder lines that were used to generate these data. Since the paper is a comparison between LRRK2-GS and LRRK2-GSDA expression pattern of both lines should be shown in Fig 1H.

3. Fig 2: provide significance levels between Control-GS/DA and GS-GS/DA groups. The statistical method should employ ANOVA instead of t-test for behavioral studies.

4. Fig 4 shows the appearance of 75+ kDA alpha-synuclein bands (oligomers) in LRRK2-GS forebrain tissue but not in LRRK2-GS/DA. However, the blot is run separately for all the genotypes. Technically, alpha-synuclein oligomer measurements should be performed comparing all the 3 genotypes from individual brain regions in a single blot.

Reviewer #2:

This is a very detailed, well written study about a new LRRK related model. The data are thorough and well presented. I have only one comment.

The authors mention the lack of nigral pathology in the present model as well as the association of LRRK2 PD with synucleinopathies. Would it not be appropriate to remind the readers that many alpha synuclein based models also lack nigral pathology, thus uniting these paradoxes.
